# Image Haze Removal Method Based on Histogram Gradient Feature Guidance

**DOI:** 10.3390/ijerph20043030

**Published:** 2023-02-09

**Authors:** Shiqi Huang, Yucheng Zhang, Ouya Zhang

**Affiliations:** 1 School of Information Technology & Engineering, Guangzhou College of Commerce, Guangzhou 511363, China; 2 School of Mechanical and Precision Instrument Engineering, Xi’an University of Technology, Xi’an 710048, China

**Keywords:** remote sensing image, haze removal, gradient feature, guided filtering, dark channel prior method

## Abstract

Optical remote sensing images obtained in haze weather not only have poor quality, but also have the characteristics of gray color, blurred details and low contrast, which seriously affect their visual effect and applications. Therefore, improving the image clarity, reducing the impact of haze and obtaining more valuable information have become the important aims of remote sensing image preprocessing. Based on the characteristics of haze images, combined with the earlier dark channel method and guided filtering theory, this paper proposed a new image haze removal method based on histogram gradient feature guidance (HGFG). In this method, the multidirectional gradient features are obtained, the atmospheric transmittance map is modified using the principle of guided filtering, and the adaptive regularization parameters are designed to achieve the image haze removal. Different types of image data were used to verify the experiment. The experimental result images have high definition and contrast, and maintain significant details and color fidelity. This shows that the new method has a strong ability to remove haze, abundant detail information, wide adaptability and high application value.

## 1. Introduction

Image data are a very important data resource, and more than 75% of the information is taken from a two-dimensional image. Image data acquisition is usually affected by various factors, such as ambient light intensity and weather conditions, which have a great impact on the image quality. Haze weather has become a typical bad weather, which has a great impact on outdoor shooting, outdoor tracking, outdoor monitoring, automatic driving and optical imaging remote sensing. Because the air contains a lot of suspended particles, aerosols and dust, the sunlight penetration rate can be significantly reduced, and the image quality obtained by the sensor terminal is degraded, which is characterized by blurred details, decreased contrast and color distortion. Therefore, removing haze from outdoor images and optical remote sensing images, and improving image quality and visual effect, has become an important part of image preprocessing.

There are many image haze removal methods, which can be divided into three categories. The first type is model-based methods. For example, Fattal proposed the method of using a Markov model to calculate color information [1], He et al. proposed the dark channel prior (DCP) method [2], Ling et al. proposed the estimation method for perceptual transmission [3], and Berman et al. proposed a nonlocal defogging algorithm using fog line to estimate the transmittance map [4]. At present, the DCP algorithm and its series of improved algorithms are still the main methods for haze removal in a single image [5,6,7,8,9,10]. The common approach of this type is to remove haze by solving or estimating the atmospheric light coefficient value and the atmospheric transmittance rate with the physical model of atmospheric scattering from different aspects. Therefore, the accuracy of parameter estimation directly affects the haze removal effect. The second type is the filtering-based method. This mainly comprises Retinex theory, homomorphic filtering, bilateral filtering, histogram processing and wavelet transform. Kim et al. proposed a local histogram equalization method to realize image enhancement [11]. Land et al. proposed the Retinex theory, and believed that the perception of the human visual system mainly comes from the reflection component of objects [12,13]. According to Retinex theory, the haze can be removed by filtering the light component and retaining the reflection component. The most representative algorithms are the single-scale Retinex (SSR) algorithm [14,15,16], the multi-scale Retinex (MSR) algorithm [17,18] and the multi-scale Retinex color restoration (MSRCR) algorithm [19]. There are many improved algorithms based on these three algorithms [20,21,22]. A single haze removal algorithm usually struggles to solve the problems of haze removal and detail preservation, so some scholars have fused image restoration and image enhancement methods to give full play to the advantages of various algorithms [23,24,25]. To protect geometric details while filtering, He et al. further proposed the guided filtering theory [26,27], and some scholars have applied this theory to image haze removal [28,29,30,31]. The third type is based on the deep learning methods that have sprung up in recent years [32,33,34,35,36,37]. Cai et al. used convolution neural network (CNN) to estimate the transmittance rate for the first time, and combined this with a traditional algorithm to remove haze from the image [33]. Ren et al. proposed using a multi-level convolution neural network to estimate the transmission [34]. Li et al. used conditional generative adversarial networks (CGAN) to remove image haze [36]. Bu et al. proposed an end-to-end network and combined the guided filtering theory to achieve image haze removal [37]. It can be seen from these studies that deep learning theories are mainly used to estimate the transmission rate in the atmospheric scattering model for image haze removal. The biggest difficulty in image haze removal based on deep learning methods comprises two aspects; one is the acquisition of training image data, the other is the design and training of the network model.

At first, the proposed image haze removal algorithms were all used for outdoor images, and then have been slowly applied to remote sensing images [20,24,38,39,40,41,42,43,44]. In a broad sense, outdoor images can also be considered as remote sensing images. However, from the perspective of the narrow professional field, remote sensing image refers to the image obtained by sensors via aerospace, aviation or near-ground carrier. Therefore, there are some differences between remote sensing images and outdoor images, mainly reflected in the following aspects. First, the platforms for image acquisition are different. Second, the resolution of the image is different. The resolution of remote sensing image is low, and the resolution of outdoor image is high. Third, the representation modes of images are different. Outdoor images only have RGB color images, while remote sensing images include gray scale, panchromatic, multispectral and hyperspectral images. Fourth, the outdoor image comprises two types, i.e., including sky or no sky area, the depth of field of the image is relatively long, and the distance between scenes is relatively large. Fifth, a remote sensing image contains the gray distribution of the Earth’s surface scene, does not contain the sky area, and the depth of field is relatively small. Therefore, if the outdoor image haze removal method is directly used to process the haze remote sensing image, although it can remove part of the influence of haze, it usually cannot achieve the desired effect in detail and color, and generally needs to be improved accordingly. The idea of the above haze image classification method is also suitable for the haze removal of remote sensing images. It can be seen from the description of relevant documents that the existing haze removal methods of remote sensing images are basically the same as natural image haze processing, which can be roughly divided into three categories, namely, model-based, filtering and deep learning. This paper also proposed a new HGFG algorithm for image haze removal, and its advantages or contributions are as follows.

(1) It has good universality and application scope. It can be used to process different types of haze images, such as color images, multispectral remote sensing images and single band gray images.

(2) Two-phase haze elimination processes were designed; the first is coarse treatment and the second is fine treatment. The saturation and brightness of the original image are enhanced and balanced, and then it is processed by a difference operation on the original haze image. In this way, the image haze rough processing is realized, that is, the first haze removal process. The gradient guided image is used to modify the estimation of atmospheric transmittance to improve the estimation accuracy of parameters. Combined with the atmospheric scattering model, the effective haze removal is further completed, i.e., the fine processing of the second haze removal process.

(3) A multidirectional gradient information extraction strategy was designed. The gradient feature of the image is extracted through multiple directions, so that the obtained gradient image can carry more abundant and complete information when it is used as the guide image, so as to improve the accuracy of parameter estimation and haze removal.

(4) In the guided process, the adaptive assignment of regularization parameters is designed. The parameters are automatically adjusted according to different input image contents, which make the processing results more consistent with the actual situation and makes the haze removal more effective.

The rest of this paper is organized as follows. In Section 2, the related theories and work are briefly introduced. The proposed HGFG algorithm is described in detail in Section 3. In Section 4, the experimental settings and the results are discussed, including different parameter settings and comparisons of different methods. Finally, Section 5 concludes this paper and outlines the future work.

## 2. Related Theory and Work

### 2.1. Atmospheric Physical Scattering Model and Dark Channel Prior Method

The model most widely used in image haze removal based on an atmospheric physical scattering model was proposed by McCartney et al. [45], and the specific mathematical model is given in Equation (1).

(1) I(x)=J(x)⋅t(x)+A⋅[1−t(x)]

where I(x) denotes the scene image acquired by the imaging device, which is the image affected by haze. J(x) denotes a haze-free image of the real scene, i.e., obtained in haze-free weather. x is the pixel, and A is the atmospheric light value, which is generally considered as a constant. t(x) is the transmittance rate, and t(x)=exp[−β⋅d(x)], where β is the dissipation coefficient of atmospheric light and d(x) is the scene depth. We adjust Equation (1) appropriately, and then Equation (2) can be obtained.

(2) $$J(x)=\frac{I(x)-A}{\max\left\{t(x),t_{0}\right\}}+A$$

To avoid the value of J(x) being too large, the lower limit of value of t(x) is usually set to lower than 0.1, i.e., $t_{0}<0.1$, so that the processed image is more in accordance with the natural scene.

It can be seen in Equation (2) that, as long as parameters A and t(x) are known, and haze image I(x) is input, then the haze-free image J(x) will be restored. If I(x) and J(x) are RGB color images, in this model, the transmittance of each pixel in R, G and B channels is basically the same. Similarly, the values of atmospheric light in these three channels are very close, and can be approximately considered to be equal.

The DCP algorithm is a typical haze removal algorithm based on an atmospheric physical scattering model, which is still widely used in haze image restoration. Through statistical analysis of a large number of outdoor clear images obtained under clear weather conditions, in most images without a sky region, there is at least one color channel, and the pixel value in the image is very low, close to zero. In general, for any clear and haze-free RGB image J(x) without sky area, its dark channel image $J^{\mathrm{dark}}(x)$ can be expressed by Equation (3).

(3) $$J^{\mathrm{dark}}(x)=\underset{y\in \Omega (x)}{\min}\left[\underset{c\in \left\{r,g,b\right\}}{\min}J^{c}(y)\right]$$

where $J^{c}$ is a gray image of any color channel in image J(x), c∈{r,g,b} is a color channel, Ω(x) is a local window centered on pixel x, and $J^{\mathrm{dark}}$ is the corresponding dark channel image.

**Figure 1:**
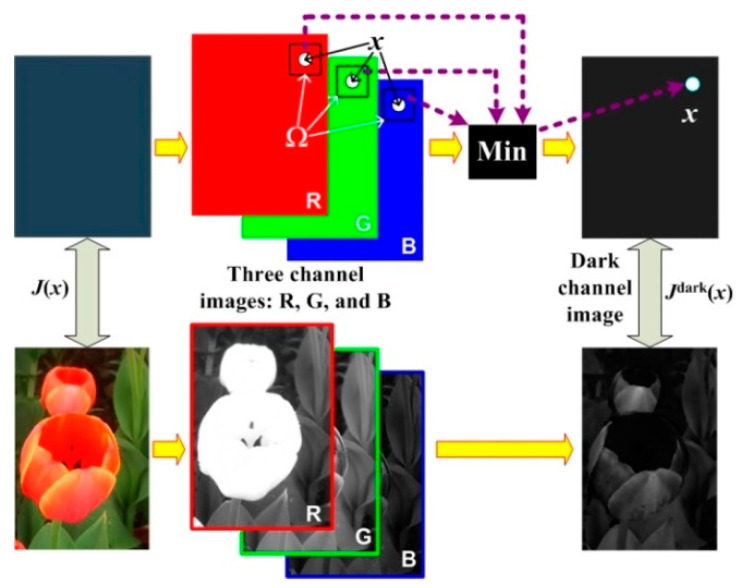
Illustration of dark channel image acquisition principle.

The principle diagram for obtaining a dark channel image of any color image J(x) is shown in Figure 1. In Figure 1, the acquisition of the dark channel image is essentially performed by minimum filtering. Firstly, three color channel images of color image J(x) are extracted, and then the minimum value of each pixel x in image J(x) is calculated in the neighborhood window of Ω(x) in the three channel images. A gray scale $J^{\mathrm{dark}}(x)$ with the same size as image J(x) is composed of all the minimum values. Through the minimum filtering operation, the white scene or the bright color region can be filtered out in the gray image of three channels. These white objects will interfere with the estimation of atmospheric light value and affect the accuracy of the estimation. The size of the minimum filter window is determined by the area size of Ω(x). Generally, if the radius of the filter is r, then the window size of Ω(x) is Ω=2×r+1.

According to the statistical analysis of a large number of clear and haze-free outdoor images in [2], the objects producing dark channel images are three-fold, including some shadows generated by various objects in the image, some objects with saturated and bright colors in the image, and some objects with black or dark colors in the image. The above three situations often pertain in a clear and haze-free image, so the dark channel prior theory is valid and applicable in most cases. Using the dark channel prior method to remove haze in images mainly comprises three processes. The first is to obtain the dark channel map of the input image. Secondly, the dark channel map is used to estimate the atmospheric light value A and the initial rough transmittance rate $\tilde{t}(x)$ in the original image. Then, the soft matting algorithm is used to refine the initial transmittance. Finally, according to the atmospheric physical scattering model shown in Equation (2), the haze-free image is restored. For a detailed implementation process and principle, please refer to reference [2,46,47].

Assuming that the transmittance in a local area Ω(x) centered on pixel x is a constant $\tilde{t}(x)$, the atmospheric light value $A^{c}$ has been obtained, and the color channel is c∈{r,g,b}. According to the principle of the prior method of the dark channel, for the haze-free image J(x), the value of all pixels in the dark channel image $J^{\mathrm{dark}}$ is very low, which is close to zero, and the estimated value of transmittance rate can be obtained, i.e.,

(4) $$\tilde{t}(x)=1-\alpha \cdot \underset{y\in \Omega (x)}{\min}\left[\underset{c}{\min}\frac{I^{c}(y)}{A^{c}}\right]$$

where α is a constant parameter. In practical applications there will always be a small amount of dust particles in the atmosphere. To make the image more suitable for the natural scene after defogging, a constant parameter α is introduced in the estimation of transmittance rate.

### 2.2. Guided Filtering

In the DCP method introduced in Section 2.1, the estimation of atmospheric transmittance is relatively rough. The key technology of image haze removal based on the dark channel prior principle is the accurate estimation of transmittance. The soft matting method is used to refine the transmission estimation [2]. Although the accuracy of transmission estimation is improved, the method is not only difficult to calculate, but is also time-consuming. To simplify the calculation and to reduce the time complexity, He et al. proposed the guided filtering theory, which is used to refine the estimation of transmittance, such that the image can save more detailed information [26,27].

Suppose I is the input image to be filtered, G is the guided image, and F is the filtered image. According to the idea of image guided filtering, there is a linear relationship between the guided image G and the output image F in the local filtering window $\omega_{k}$, and the relationship model between them can be expressed by Equation (5).

(5) $$F_{i}=a_{k}G_{i}+b_{k},\;\forall i\in \omega_{k}$$

Here $a_{k}$ and $b_{k}$ are linear coefficients, and they are constants in the filter window $\omega_{k}$. $\omega_{k}$ is the filtering window with pixel k as the center, and its filtering radius is r. The values of coefficients $a_{k}$ and $b_{k}$ are calculated by Equation (6).

(6) $$\left\{ \begin{array}{l} a_{k}=\frac{\frac{1}{N_{\omega }}\sum_{i\in \omega_{k}}G_{i}I_{i}-\mu_{k}{\bar{I}}_{k}}{\sigma_{k}^{2}+\varepsilon }\\ b_{k}={\bar{I}}_{k}-a_{k}\mu_{k} \end{array}\right.$$

where $\mu_{k}$ and $\sigma_{k}^{2}$, respectively, represent the mean and variance of the guided image G in the local filtering window $\omega_{k}$. $N_{\omega }$ is the total number of all pixels in filter window $\omega_{k}$. ${\bar{I}}_{k}$ is the average value of the input image I in the corresponding window. ε is the regularization parameter.

In the process of processing, as window $\omega_{k}$ moves, pixel i will be included in the multiple different windows $\omega_{k}$ covering it [48]. Because the values of pixels in different windows are different, the values of coefficients $a_{k}$ and $b_{k}$ in each window calculated by Equation (6) are also different. To make the values of $a_{k}$ and $b_{k}$ more accurate, it is necessary to sum and average the corresponding values obtained in all the windows $\omega_{k}$ containing pixel i, and the mathematical model is as follows.

(7) $$\left\{ \begin{array}{l} {\bar{a}}_{k}=(\sum_{k\in \omega_{k}}a_{k})/N_{\omega }\\ {\bar{b}}_{k}=(\sum_{k\in \omega_{k}}b_{k})/N_{\omega } \end{array}\right.$$

At the same time, Equation (5) is rewritten as follows.

(8) $$F_{i}={\bar{a}}_{k}G_{i}+{\bar{b}}_{k},\;\forall i\in \omega_{k}$$

In the principle of guided filtering, the window size r and regularization parameter ε are two very important parameters, and their values will directly affect the final filtering results. The larger the filter window radius r is, the more obvious the smoothing effect is; the smaller the filter window is, the more details are kept. The larger the regularization parameter ε is, the smaller the coefficient $a_{k}$ is, and the smaller the edge information of the guide image G is kept. On the contrary, the larger the coefficient $a_{k}$ is, the more information of the guide image is contained in the output image. Since the regularization parameter ε is a constant, and the same local linear model is used in filtering the whole image, halo artifacts may appear in the area where the pixel gray value changes greatly in the filtered image. To solve the problem of guided filtering, some scholars have proposed a series of improved guided filtering algorithms, such as weighted guided filtering [49], gradient field guided filter [50], alternating guided filter [51], dynamic guided filter [52,53] and adaptive guided filter [54].

## 3. Principle Description of HGFG Algorithm

**Figure 2:**
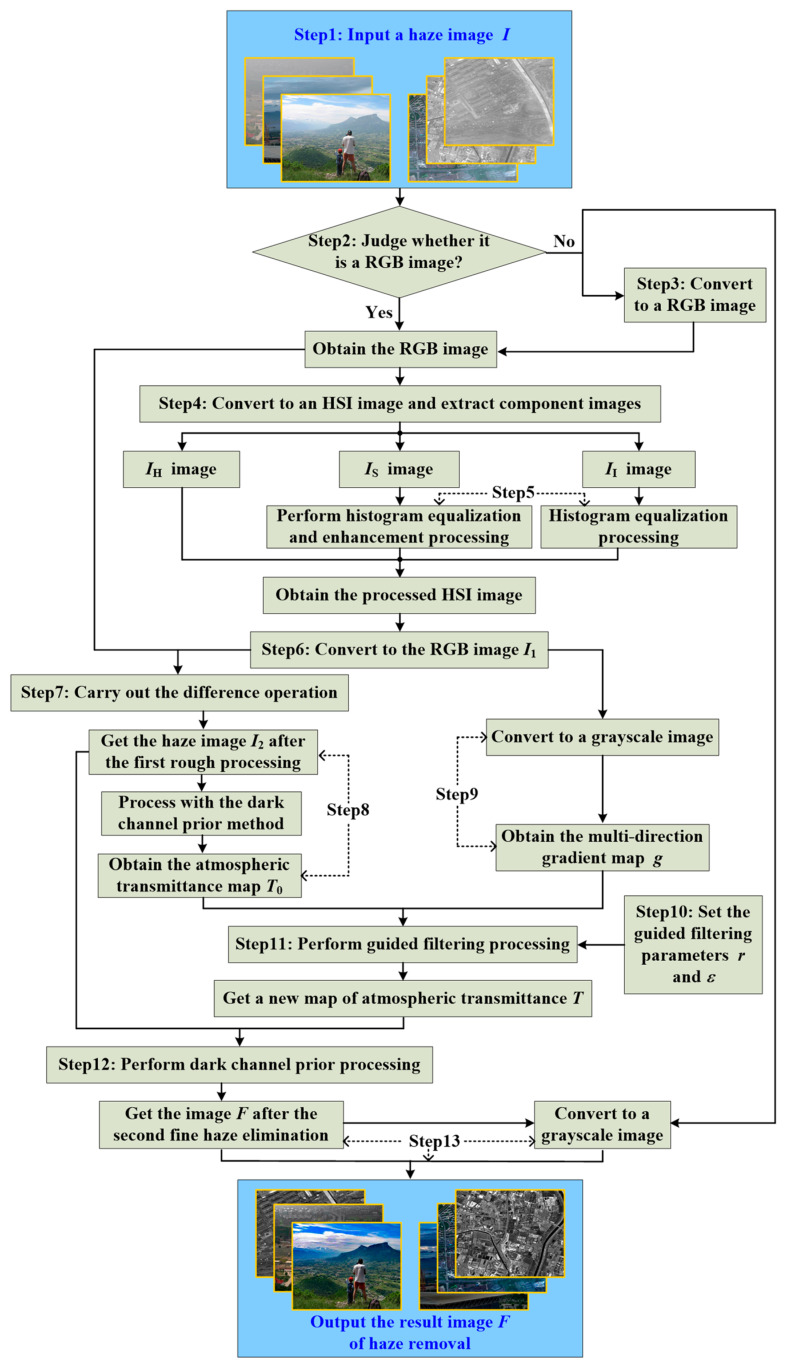
Flow diagram of HGFG algorithm.

This paper proposes an image haze removal algorithm based on histogram gradient feature guidance (HGFG), which organically combines the guiding filtering principle and dark channel prior method, and fully considers the content and characteristics of the image. Therefore, its outstanding feature is that it has good universality, can effectively eliminate image haze, has almost no halo and gradient artifacts, and can maintain good clarity and contrast. The principle block diagram of the HGFG algorithm is shown in Figure 2, and its main implementation steps are as follows.

(1) Input the original haze image I. The input image is a haze image obtained under hazy weather, so it is affected by the haze weather, resulting in the graying of the image and the blurring of its details. The input image may be outdoor scenes, multi-spectral remote sensing images and single-band gray sensing image images.

(2) Judge whether the input image is an RGB color image. Outdoor images are usually RGB color images, but for remote sensing images, there are single-band gray images. If it is a gray image, go to Step (3); if it is a color image, go directly to Step (4).

(3) Convert the single-band gray images or panchromatic images into color images. At present, the image haze removal algorithms are mainly proposed for color image processing, such as the typical DCP algorithm, which is specially designed for RGB images. Therefore, the input gray remote sensing image needs to be converted into a false color image, and the conversion formula is shown in Equation (9).

(9) $$\left\{ \begin{array}{l} RGB(:,:,1)=R=a\times I\\ RGB(:,:,2)=G=b\times I\\ RGB(:,:,3)=B=c\times I \end{array}\right.$$

where *R*, *G* and *B*, respectively, represent the three channels of a color image, I denotes the input single-band gray image, and a, b and c, respectively, represent the coefficients multiplied by the gray image I. Because the DCP algorithm requires that there is a minimum value in the three channel images, if it is not multiplied by different coefficients, then it cannot be processed by the DCP algorithm. Therefore, there must be some difference between the three values. Here, let a=1.1, b=1.0, and c=0.9.

(4) The RGB image is converted into the HSI model image, and three component images H, S and I are extracted, as represented by $I_{H}$, $I_{S}$ and $I_{I}$, respectively. The $I_{H}$ image contains the types and attributes of colors, the $I_{S}$ image represents the saturation of colors, and the $I_{I}$ image represents the brightness of pixel values.

(5) The $I_{S}$ and $I_{I}$ component images are processed. From the previous analysis, it can be seen that although the haze image in the same scene has a similar hue as the real clear image, its color is in an unsaturated state, and its average brightness value is a bit dark. The similarity of hue indicates that there is little difference in the attributes or types of colors, so this does not need to be corrected, otherwise it will cause serious color distortion. Low saturation indicates that the color of the image is lighter, and the color is darker when it is oversaturated. Therefore, in the HGFG algorithm, the $I_{S}$ image will be enhanced and histogram equalization processing will be performed, which enhances the color saturation. Due to the existence of image haze, the reflection value of the image increases. In fact, the brightness of the image is not bright enough. Therefore, to balance and enhance the brightness value of the image, the intensity of the global reflected light can be reduced, so as to preliminarily eliminate the haze and improve the brightness of the scene. So the image $I_{I}$ is equalized to achieve the purpose of enhancement.

(6) The processed HSI image is converted to the RGB image $I_{1}$.

(7) After the first haze elimination, the image $I_{2}$ is obtained. The difference between the original RGB image I and the new RGB image $I_{1}$ obtained in Step (6) is calculated, and the absolute value of the difference is taken to obtain the image $I_{2}$ after the first preliminary haze processing.

(8) The initial atmospheric transmission $T_{0}$ is obtained. The DCP algorithm is used to process the image obtained in Step (7), and the corresponding atmospheric transmittance rate $T_{0}$ is extracted.

(9) The multidirectional gradient image G of image $I_{1}$ is obtained. Firstly, the image $I_{1}$ is transformed into a gray image, and then the gradient feature map of the gray image is extracted from multiple directions. For a two-dimensional image f(x,y), its change rate at pixel (x,y) is defined as the gradient. A gradient is a vector whose magnitude is usually defined by the model shown in Equation (10).

(10) $$G\left[f(x,y)\right]=\sqrt{{\left(\frac{\partial f}{\partial x}\right)}^{2}+{\left(\frac{\partial f}{\partial y}\right)}^{2}}=\sqrt{{\left(G_{x}\right)}^{2}+{\left(G_{y}\right)}^{2}}$$

where $G_{x}$ and $G_{y}$ represent the horizontal and vertical gradients, respectively. For digital images, the gradient is discrete, that is, the differential operation is replaced by the difference operation. The first-order partial derivative finite difference method in the 3×3 neighborhood is used to calculate the gradient of the image. To enhance the gradient information, the corresponding weight is increased; for example, the weight coefficient becomes two. Similarly, only the gradient information is obtained from the horizontal and vertical directions, and the gradient information in the diagonal direction is ignored. Therefore, in the HGFG algorithm, multiple directions are set to extract gradient information, which makes the extracted gradient information more complete and accurate. The specific calculation formula is shown in Equation (11).

(11) $$\left\{ \begin{array}{l} g_{0}=f(x+1,y-1)+2f(x+1,y)+f(x+1,y+1)-f(x-1,y-1)-2f(x-1,y)-f(x-1,y+1)\\ g_{45}=f(x-1,y)+2f(x-1,y+1)+f(x,y+1)-f(x,y-1)-2f(x+1,y-1)-f(x+1,y)\\ g_{90}=f(x-1,y+1)+2f(x,y+1)+f(x+1,y+1)-f(x-1,y-1)-2f(x,y-1)-f(x+1,y-1)\\ g_{135}=f(x,y+1)+2f(x+1,y+1)+f(x+1,y)-f(x-1,y)-2f(x-1,y-1)-f(x,y-1) \end{array}\right.$$

In Equation (11), $g_{0}$, $g_{45}$, $g_{90}$ and $g_{135}$ represent the first-order derivative differentials in the $0^{0}$, $45^{0}$, $90^{0}$ and $135^{0}$ directions, respectively, and we use them to calculate the horizontal and vertical gradients. After obtaining the gradient information in the above four directions, the horizontal and vertical gradients are calculated by Equation (12).

(12) $$\left\{ \begin{array}{l} G_{x}=g_{0}+\sqrt{2}(g_{45}+g_{135})/2\\ G_{y}=g_{90}+\sqrt{2}(g_{135}-g_{45})/2 \end{array}\right.$$

(10) Set parameters r and ε for the guided filter processing. In the guided filtering, there are two very important parameters, namely, filter radius r and regularization parameter ε. Their different values will affect the filtering results. In the HGFG algorithm, the size of filter radius is set to 32, i.e., r=32. The value of the regularized parameter ε is set to adaptive update, and its specific mathematical definition is shown in Equation (13).

(13) $$\varepsilon =\sigma_{}^{2}/L$$

where $\sigma_{}^{2}$ and L are the variance and gray level of the guide image G, L=max(G)−min(G).

(11) The input image is guided and filtered, and a new atmospheric transmittance rate T is obtained. The initial atmospheric transmittance map $T_{0}$ and gradient characteristic rate g are processed by guided filtering, and a new atmospheric transmittance map T can be obtained.

(12) The second haze elimination fine processing procedure is carried out for image $I_{2}$. Using the DCP algorithm and the new atmospheric transmittance rate T to remove haze from the coarse processed image $I_{2}$, the restored haze image F can be obtained.

(13) The processing result, image F, is output. If the input image I to be processed is a color image, the resulting image F is directly output. If the input image I to be processed is a gray image, it is necessary to convert the processing result image F into a gray image, and then output the final result.

## 4. Experimental Results and Analysis

The biggest advantage of the proposed HGFG algorithm is that it can effectively remove the haze from the image, and it has good universality. To verify the advantages and feasibility of this approach, this section designs experiments of different scene images, and carries out comparative experiments with different methods. The experimental data are introduced in each group experiment. The experimental methods include the HGFG algorithm, the dark channel prior (DCP) method [2], the guiding filter (GF) algorithm [27], the single-scale Retinex (SSR) algorithm [15], the DeHazeNet algorithm with deep learning [33] and the histogram equalization (HE) algorithm. In the following experiment, the scale parameter of the SSR algorithm is set to 128, and the filter window size of the GF algorithm is set to 32.

### 4.1. Haze Removal Experiment of Outdoor Images

**Figure 3:**
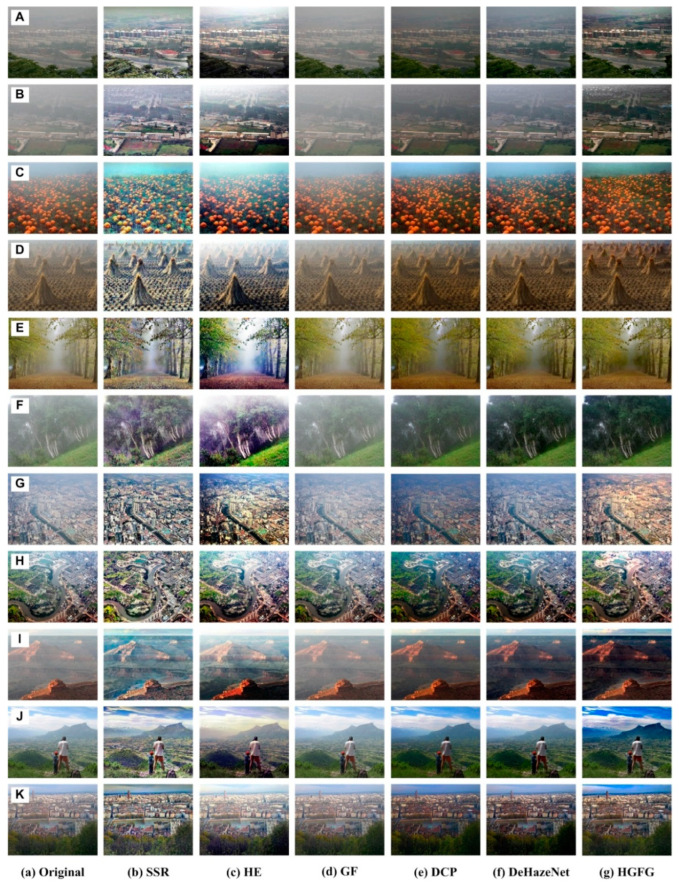
Experimental results of haze removal in outdoor images. ((**A**)–(**K**) represent different types of haze images; a represents original images, (**b**)–(**g**) represent the resulting images processed by the SSR, HE, GF, DCP, DeHazeNet and HGFG algorithms, respectively).

The data used in this experiment are shown in Figure 3a. These images are outdoor photos taken with cameras, which were affected by haze to varying degrees, and they come from images published on the internet. The images shown in Figure 3A–K, respectively, represent outdoor images of different scenes. The scene shown in Figure 3A,B is a village with houses and various natural landscapes. The images shown in Figure 3C,D are agricultural land. Figure 3E,F,I,J are natural scene images, which contain trees and grasslands. The scene shown in Figure 3G,H,K is an urban area. The distant part of the images shown in Figure 3I–K contains the sky region, while the rest of the images (Figure 3A–H) do not contain the sky region. The above six methods were used to remove haze from these images, respectively. The experimental results are shown in Figure 3b–g, obtained by the methods SSR, HE, GF, DCP, DeHazeNet and HGFG in turn.

It can be seen in Figure 3b that, although the SSR algorithm can reduce the influence of haze and remove part of the haze, the processed image is a bit bright in hue, and the color is seriously distorted. The SSR method involves the value of scale parameters, and different parameter values will have different results. For specific cases, please refer to the literature [24]. The experimental results shown in Figure 3c were obtained by the HE method. The essence of this method is to adjust the uniformity of the brightness value of the image, so there is no haze removal. Due to the adjustment of the brightness value, the spatial distribution is more balanced, which leads to the improvement of the visual effect. This method of removing haze is not ideal, the result is bright, and the color has a certain degree of distortion. The haze removal result of the GF algorithm is shown in Figure 3d, and its effect is not good. The initial purpose of the GF algorithm is to filter the noise and keep the edge. However, in a sense, haze is not noise, so the effect of haze image processing with the GF algorithm alone is poor. The results shown in Figure 3e–g have been processed by DCP, DeHazeNet and HGFG, respectively. Their processing effects are relatively close, and in this group of experiments, they are the best. The overall effect is that the HGFG algorithm is better than the other two methods, while the DCP algorithm is slightly better than the DeHazeNet method. For the DeHazeNet algorithm, the selection of training data set will affect the final haze removal effect, which is a common defect of deep learning and other machine learning algorithms.

Most outdoor images contain sky regions, but many algorithms do not deal with the sky region well. The HGFG algorithm can effectively process these outdoor images containing sky regions, which is shown in Figure 3I(g)–K(g). It can be seen in Figure 3 that the HGFG algorithm can not only obtain clear ground objects, but can also deal with the sky region with a far depth of field. For example, in the image of an urban area shown in Figure 3K, the urban area obtained by other algorithms is not very clear, while the result obtained by the HGFG algorithm is the clearest, as shown in Figure 3K(g). Secondly, the DCP algorithm and DeHazeNet algorithm are better. The SSR algorithm and HE algorithm have some certain haze removal abilities, but they are not ideal for dealing with the sky area. The GF algorithm has the worst processing ability, achieving almost no processing.

After the above analysis, the HGFG algorithm is deemed the best in removing haze from outdoor images. Not only is the haze elimination relatively clean, but the detail information is also maintained more effectively, and the color distortion effect is also the least noticeable. Detailed quantitative analyses and comparisons will be carried out in Section 4.3.

### 4.2. Haze Removal Experiment of Remote Sensing Images

**Figure 4:**
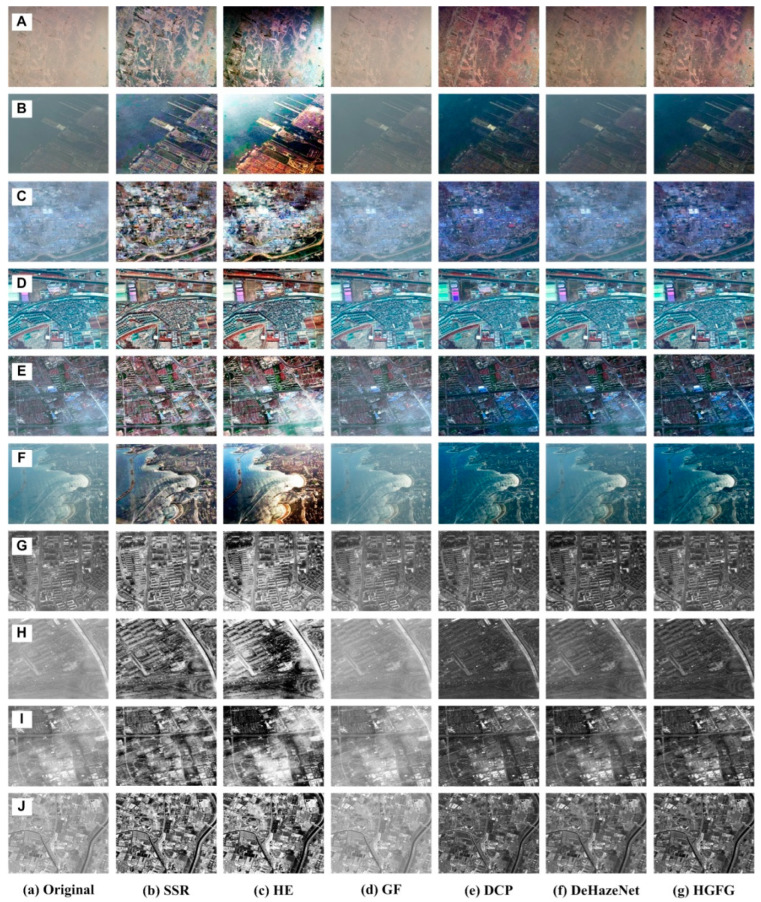
Experimental results of haze removal of remote sensing images. ((**A**)–(**J**) represent different types of haze images; a represents original images, (**b**)–(**g**) represent the resulting images processed by the SSR, HE, GF, DCP, DeHazeNet and HGFG algorithms, respectively).

Optical remote sensing images are a very important data source, and have been widely used in many fields. However, optical remote sensing imaging occurs in the visible spectral range, which will be affected by haze weather, so the purpose of this section is to discuss the restoration of remote sensing haze images. Optical remote sensing images usually include multispectral images, panchromatic images and single-band gray images. Multispectral images are RGB color images synthesized with any three single-band images. The images shown in Figure 4a are the original haze remote sensing images. The image shown in Figure 4A is a QuickBird image with a spatial resolution of 2.5 m and a size of 600 × 600. Figure 4B and Figure 4F are UAV images, their spatial resolution is 2 m, and the image sizes are 595 × 393 and 500 × 350, respectively. Figure 4C and Figure 4E are GeoEye-1 images with a spatial resolution of 2 m, and their sizes are 700 × 700 and 1000 × 1000, respectively. Figure 4D is a Worldview-3 image with 1.24 m resolution and 1000 × 1000 size. The images shown in Figure 4G,H are GeoEye-1 images. Figure 4G is a single-band gray image with 2 m resolution and 1000 × 1070 size. Figure 4H is a panchromatic grayscale image, its resolution is 0.5 m and its size is 1000 × 1000. The images shown in Figure 4I,J are also Worldview-3 images with a spatial resolution of 1.24 m and sizes of 1024 × 1024. These images were affected by haze to varying degrees, resulting in poor visual effects and blurred details.

For all remote sensing images, the effect of removing haze using the SSR algorithm is good. However, for the color haze image, the color of the processed image is a little distorted and bright, as shown in Figure 4b. At the same time, the SSR algorithm is more useful for the removal of haze in gray remote sensing images, because it does not involve the problem of color distortion.

In Figure 4B,F, a large number of the imaging scenes include water areas. The water area in Figure 4B is static, but in Figure 4F it is dynamic. For water scene images with large areas, many algorithms are not very effective. For example, the results of using the HE algorithm to process these two images are not ideal, as shown in Figure 4B(c),F(c). In Figure 4, the best result of the HE algorithm is Figure 4D(c). For other images, its processing effect is not good. The main reason is that the haze in the image shown in Figure 4D is uniform, which is just suitable for histogram processing, so the effect is better, while the haze in other remote sensing images is uneven. For gray remote sensing images, the image processed by the HE algorithm is a little too bright, and the area with more haze presents as white, as shown in Figure 4G(c)–J(c).

It can be seen in Figure 4d that for all remote sensing images, the GF algorithm has a poor ability to remove haze, and hardly does so. This shows that the GF algorithm has a low ability to eliminate haze in remote sensing images. It is also proven that the GF algorithm is only useful for image noise removal, especially Gaussian noise removal, but for image haze removal, its ability is very limited.

Among the remaining three methods, the DeHazeNet method has a weaker ability to remove haze, especially for the two water area images (Figure 4B,F) and the uneven haze images (Figure 4C,E,I). The reason is related to the selection of training data. The algorithm with a better haze removal ability is the DCP algorithm, as shown in Figure 4e. The DCP algorithm can only process color images, but in this experiment, these gray images are converted into color images according to Equation (9), and then they are again converted into gray images after being processed. Although this method can effectively smooth the haze in the image, halos and artifacts often arise. For example, in Figure 4A(e),F(e), an obvious halo phenomenon occurs in the remote sensing image processed by the DCP algorithm. The best method of processing is the HGFG algorithm. It has good experimental results—not only is the haze removal obvious, but the clarity and visual effect are also good, the contrast has been improved, and the distortion of the image is very small, as shown in Figure 4g.

### 4.3. Quantitative Evaluation and Analysis

The previous evaluation of the experimental results is performed from the visual point of view, which is related to personal experience and visual judgment criteria. Therefore, we will use some objective evaluation parameters to evaluate the performance. The common parameters are information ratio (IR), structure similarity (SSIM), peak signal-to-noise ratio (PSNR) and contrast ratio (CR). The purpose of removing haze from the image is to restore an image without haze, that is, an image without haze and sunny weather. Therefore, while removing haze, it is necessary to keep the geometric details and the amount of information in the image as closely as possible, so as to obtain a clearer image, i.e., an image with higher contrast. Because we are evaluating the haze removal effect, we compare between the haze-removed image and the original haze image. If more haze is removed and more details are kept, the performance of the algorithm is better. For the definition and calculation of SSIM and PSNR, refer to Ref. [55]. Next, only the IR and CR parameters are discussed.

Image information ratio (IR) refers to the amount of information available to compare between two images. The average information of an image is generally described by information entropy, so the mathematical definition of the *IR* is shown in Equation (14).

(14) $$IR=H_{F}/H_{O}$$

where $H_{F}$ and $H_{O}$ represent the information entropy of the haze-removed image and the original haze image, respectively. The larger the IR value is, the better the haze removal effect is, and it will contain more information.

The structural similarity (SSIM) parameter describes the similarity between the contours, edges and details of two images. Because the haze-removed image is compared with the original image, the more effective the haze removal is, the greater the difference will be between the images, and the smaller the SSIM value will be. If the haze removal effect is worse, the difference between will smaller, and the images will be more similar, so the SSIM value will be larger. When the two images are exactly the same, the SSIM value is equal to one.

After the image is affected by haze, the effect is equivalent to adding noise. Therefore, the peak signal-to-noise ratio (PSNR) parameter of the image can be used to describe the problems of haze removal. When the haze removal effect is better, the PSNR value will be smaller. If the processed image is closer to the original image, and particularly when it is the same as the original image, the value of the PSNR will be infinite.

Image clarity can be described by the parameter contrast ratio (CR). When the *CR* value is higher, the image is clearer and more hierarchical. The calculation formula of image contrast ratio is as follows:

(15) $$CR=\sum_{\delta }{\left[\delta (i,j)\right]}^{2}P_{\delta }(i,j)$$

where i and j represent the gray values of adjacent pixels, δ(i,j)=|i−j| denotes the gray difference between adjacent pixels, and $P_{\delta }(i,j)$ is the probability of gray difference |δ(i,j)| between adjacent pixels.

**Figure 5:**
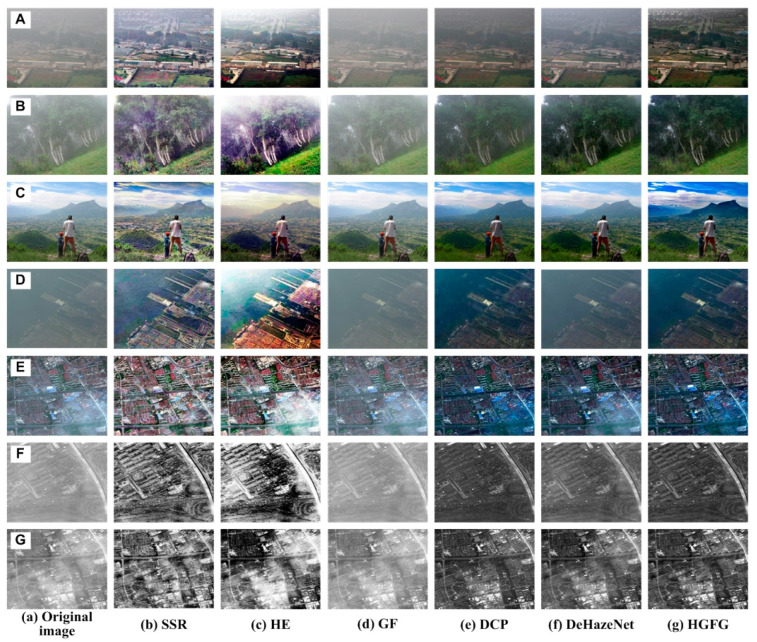
Images for quantitative analysis ((**A**)–(**G**) represent different types of haze images; (**b**)–(**g**) represent the resulting images processed by the SSR, HE, GF, DCP, DeHazeNet and HGFG algorithms, respectively).

**Figure 6:**
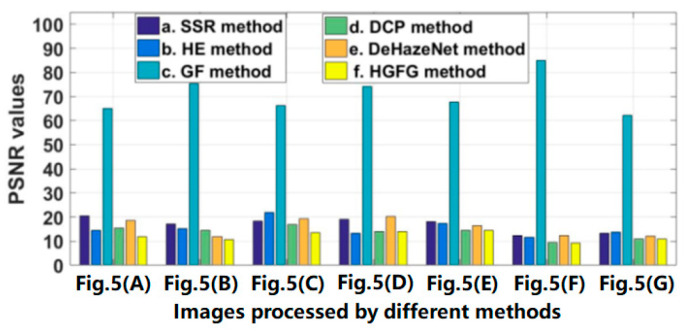
The PSNR values of the images shown in Figure 5.

**Figure 7:**
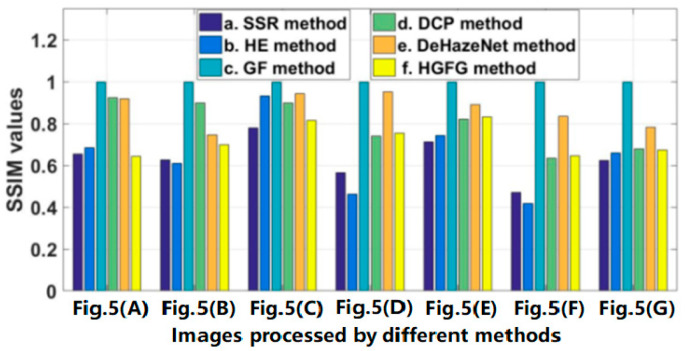
The SSIM values of the images shown in Figure 5.

**Figure 8:**
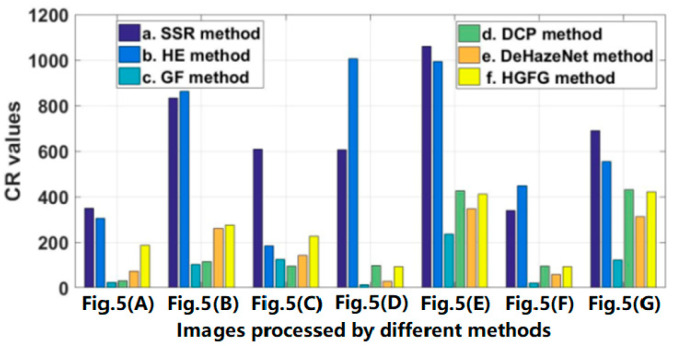
The CR values of the images shown in Figure 5.

**Figure 9:**
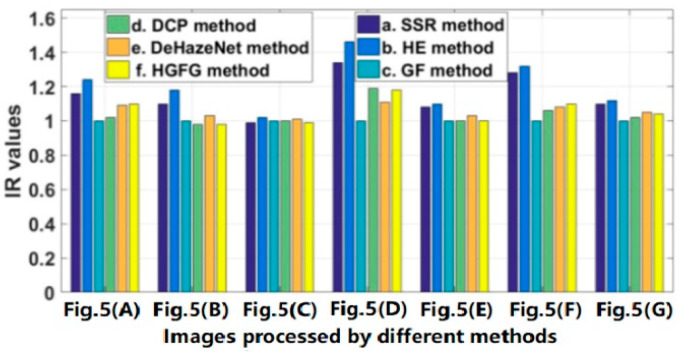
The IR values of the image shown in Figure 5.

The data of some images and their experimental results have been selected from a previous series of experiments to analyze quantitatively, and their evaluation parameters have been calculated. The images and results used for quantitative analysis are shown in Figure 5. Figure 5A–G are different original haze images, and Figure 5b–g represent the resulting images processed by SSR, HE, GF, DCP, DeHazeNet and HGFG, respectively. At the same time, the values of the four evaluation parameters—IR, SSIM, PSNR and CR—of these images have been calculated, respectively. For the convenience of comparative analysis, the calculated parameter data have been visualized, as shown in Figure 6, Figure 7, Figure 8 and Figure 9. The parameter data of some images are listed in Table 1.

Figure 6 shows the PSNR value of the image shown in Figure 5. The abscissa represents the image processed by different methods, and the ordinate is the PSNR value. It is clear in Figure 6 that no matter which image is processed by the GF algorithm, its PSNR value will be much larger than those of other methods, which indicates that the GF algorithm has a poor ability to remove haze, and the processed image still retains a lot of haze interference information. For the other five algorithms, their PSNR values show little difference. However, regardless of which image is used, the PSNR value of the image processed by the HGFG method will be relatively small, which indicates that the method has a strong ability to remove haze and can eliminate the influence of haze as much as possible.

The structural similarity values of the processed images shown in Figure 5 are displayed in Figure 7. Because structural similarity is the value obtained by comparison with the original haze image, the greater the difference from the original image is, the smaller the SSIM value is. If the opposite pertains, the SSIM value will be larger. When the original image itself is achieved, the SSIM value will be the largest and equal to one. In Figure 7, the SSIM values of the SSR algorithm and the HE algorithm are relatively small, indicating that the difference between them and original image is large. In the previous analysis, it can be seen that their effects of haze removal are not the best, and their SSIM values will not be the lowest from the perspective of haze elimination. However, in fact, their SSIM values are indeed the smallest, which indicates that there is a great difference between the processed image and the original image in structure. There is only one reason for this. When they deal with the haze image, they adjust the gray spatial distribution of the original image, enhance the sense of the gray level, and change some of the details of the original image. Because the GF algorithm has a poor ability to eliminate haze, there is good similarity between the processed image and the original image, and its SSIM value is close to or equal to one. Among the other three methods, the SSIM value of the HGFG algorithm is smaller, which shows that it can remove haze and achieves great difference from the original image.

Figure 8 shows the contrast ratio parameter values of the processed images in Figure 5. Because the SSR algorithm and the HE algorithm adjust the spatial distribution structure of the image’s gray value while removing haze, the gradient information or gray level sense of the image is enhanced, and the CR value of the image processed in this way is very large. In the rest of the algorithms, the CR value of the HGFG algorithm is higher. Secondly, the CR values of the DCP algorithm and the DeHazeNet algorithm are higher, but their values are very unstable, and are sometimes high and sometimes low, indicating that their processing effects on different images are different. The minimum CR value is obtained by the image processed by the GF algorithm. Through the quantitative analysis performed in this experiment, it is shown that the HGFG algorithm can also obtain good contrast information, i.e., good clarity of the image.

As can be seen in Figure 9, aside from the SSR algorithm and the HE algorithm, the information ratios of the algorithms are close to one, which indicates that the average information of the images processed by them does not increase or decrease significantly. On the contrary, in most cases, the IR values of the SSR algorithm and HE algorithm are more than one, which indicates that more information is added, and the scene information without haze is not necessarily yielded.

The evaluation parameter data of some images are shown in Table 1. The rules reflected by them have been described in detail in Figure 6, Figure 7, Figure 8 and Figure 9, and the specific values are given here. It can be seen in Table 1 that with the GF algorithm, the SSIM value and IR value are equal to one, while the PSNR value is the largest and the CR value is almost the smallest. For example, in Figure 5A(d), showing an image processed by the GF algorithm, the maximum value of the PSNR is 65.18, and the minimum value of the CR is 24. In each processed image, the SSIM values of the SSR algorithm and the HE algorithm are relatively small, and the IR and PSNR values are relatively large, but the CR value are very large. For example, in Figure 5E, the CR values of the SSR algorithm and the HE algorithm are 1060 and 994, respectively, which are much larger than those of other methods. For the DCP algorithm and the DeHazeNet algorithm, no obvious trend emerges in processing different images, but their SSIM values are usually relatively high. For example, in Figure 5A, they are 0.9230 and 0.9174, respectively, which are higher than other methods. Although the CR value of the HGFG algorithm is not the highest, the PSNR value is the smallest in each image, and the SSIM value is relatively small.

After the above visual and quantitative analyses, we can draw the following conclusions. Although the SSR algorithm and the HE algorithm can effectively remove the haze from the image, the processed image shows an obvious phenomenon of brightness and color distortion; although the contrast is significantly improved, the average amount of information is increased, which may alter the spatial distributions of some details. The GF algorithm performs poorly in haze removal, and it is not suitable for directly processing a hazy image. The DCP algorithm and DeHazeNet algorithm can generally reduce the impact of haze, but the effects of different images are different, indicating that their universality is poor. The HGFG algorithm can usually effectively remove haze, and can process different images; its effect is good, and the consistency between the restored image and the original image’s information is better.

## 5. Conclusions

Hazy weather reduces the quality of optical images, so removing haze, and thus improving image quality and visual effect, has become an important part of image preprocessing. In this paper, according to the characteristics of the image, through the dark channel prior theory and guided filtering theory combined with the image statistics and gradient information, the HGFG algorithm was proposed. Through practical image verification, we see that the new method is effective; not only can it effectively reduce the impact of haze and improve the image clarity, but it also achieves a wide range of adaptability and less distortion. For dense and uneven haze, the removal effect is not ideal. Therefore, the next step is to study the removal of dense haze, and the fidelity of the color image.

## Figures and Tables

**Table 1:** Parameter values of some images processed by different methods.

Images	Parameter	SSR	HE	GF	DCP	DeHazeNet	HGFG
Figure 5A	SSIM	0.6551	0.6858	1.00	0.9230	0.9174	0.6434
	PSNR	20.42	14.56	65.18	15.44	18.47	11.89
	IR	1.16	1.24	1.00	1.02	1.09	1.10
	CR	349	304	24	32	74	186
Figure 5C	SSIM	0.7779	0.9307	1.00	0.8991	0.9420	0.8152
	PSNR	18.23	66.34	21.88	16.84	19.37	13.44
	IR	0.99	1.02	1.00	1.00	1.01	0.99
	CR	609	185	124	95	141	225
Figure 5E	SSIM	0.7132	0.7421	1.00	0.8212	0.8903	0.8313
	PSNR	18.21	17.34	67.68	14.49	16.52	14.49
	IR	1.08	1.10	1.00	1.00	1.03	1.00
	CR	1060	994	235	426	347	410
Figure 5G	SSIM	0.6232	0.6589	1.00	0.6798	0.7808	0.6749
	PSNR	13.24	13.78	62.30	10.91	12.20	10.79
	IR	1.10	1.12	1.00	1.02	1.05	1.04
	CR	690	555	122	431	313	421

## Data Availability

Data are available on request from the corresponding author.
